# The effect of repetitive and Deep Transcranial Magnetic Stimulation on quantitative electroencephalography in major depressive disorder

**DOI:** 10.3389/fpsyt.2024.1473743

**Published:** 2025-01-06

**Authors:** Reyhan Ilhan, Mehmet Kemal Arikan

**Affiliations:** ^1^ Department of Psychiatry, Kemal Arıkan Psychiatry Clinic, Istanbul, Türkiye; ^2^ Neuroscience Program, Graduate School of Health Sciences, Uskudar University, Istanbul, Türkiye; ^3^ Department of Mental Health and Diseases, Faculty of Medicine, Uskudar University, Istanbul, Türkiye

**Keywords:** EEG, TMS, MDD, delta, theta, alpha, beta, gamma

## Abstract

**Background:**

F-8-coil repetitive transcranial magnetic stimulation (rTMS) and H-1-coil deep repetitive transcranial magnetic stimulation (dTMS) have been indicated for the treatment of major depressive disorder (MDD) in adult patients by applying different treatment protocols. Nevertheless, the evidence for long-term electrophysiological alterations in the cortex following prolonged TMS interventions, as assessed by quantitative electroencephalography (qEEG), remains insufficiently explored. This study aims to demonstrate the qEEG-based distinctions between rTMS and dTMS in the management of depression and to evaluate the potential correlation between the electrophysiological changes induced by these two distinct TMS interventions and the clinical improvement in depressive and anxiety symptoms.

**Methods:**

A total of 60 patients diagnosed with treatment resistant depression received rTMS (n = 30) or dTMS (n = 30) along with their usual treatments in Kemal Arıkan Psychiatry Clinic. All the participants underwent resting-state qEEG recording before and at the end of 30 sessions of TMS treatment. The significant qEEG changes were then tested for their correlation with the improvement in depression and anxiety.

**Results:**

After the course of rTMS and dTMS a considerable reduction is seen in the severity of depression and anxiety. Although improvements in depression and anxiety were observed in both TMS groups, specific neural activity patterns were associated with better outcomes in depression. Patients who exhibited lower alpha activity in the left fronto-central region and higher gamma activity in the right prefrontal region following rTMS showed more significant improvements in depression symptoms. Similarly, those whose beta activity increased in the left prefrontal region but decreased in the right prefrontal region after rTMS tended to have greater reductions in depression and anxiety severity. For patients in the dTMS group, those who demonstrated a decrease in left temporal theta activity after treatment were more likely to experience a substantial improvement in depression severity.

**Conclusion:**

Following 30 sessions of rTMS with a F8 coil and dTMS with an H1 coil, notable alterations in qEEG activity with clinical significance were discerned. The persistence of these changes should be investigated in the subsequent follow-up period.

## 1 Introduction

Major Depressive Disorder (MDD) stands out as a leading global burden among psychiatric disorders (1). According to the World Health Organization (WHO) estimates, approximately 10% of the population in developing countries has reported a lifetime history of depression (1). Global Burden of Disease study data from 2019 indicates that over 300 million individuals worldwide are affected by depression (2). However, during the COVID-19 pandemic, there has been a significant increase of 27.6% in depression and 25.6% in anxiety disorders within psychiatric conditions (3).

In the treatment of MDD, second-generation antidepressants (selective serotonin reuptake inhibitors-SSRIs and serotonin-norepinephrine reuptake inhibitors-SNRIs) and scientifically proven effective psychotherapies are recommended (4). A meta-analysis has robustly demonstrated that the combination of primary care psychotherapy and pharmacotherapy is more effective than individual use (5). However, only 40-60% of depression patients respond to antidepressants/psychotherapy and remaining asymptomatic (5–9). Among them, 15–30% meet the criteria for treatment-resistant depression (TRD), characterized by a lack of response to at least two antidepressant treatments (10, 11).

Given the high prevalence of treatment resistance in depression, non-invasive neuromodulation and neurostimulation techniques have emerged as promising alternatives to traditional treatments. Among these, repetitive Transcranial Magnetic Stimulation (rTMS) with an F8 coil and Deep Transcranial Magnetic Stimulation (dTMS) with an H1 coil protocol received approval from the Food and Drug Administration (FDA) in 2008 and 2013, respectively, for treatment resistant depression (12, 13). TMS functions by generating a strong, brief magnetic field through a coil placed near the scalp, inducing an electric current in targeted brain regions. This current modulates neural activity through localized neuronal depolarization (14). Depending on the frequency and intensity of the pulses, TMS can either stimulate or inhibit neural circuits. High-frequency stimulation typically enhances neural activity, whereas low-frequency stimulation suppresses it (15).

While both rTMS and dTMS share the same basic mechanism, their coil designs lead to differences in their effects. The F-8 coil generates a focused electric field, reaching a depth of 1–1.5 cm, targeting superficial cortical regions. In contrast, the H-1 coil, developed by BrainsWay for deep TMS (dTMS), produces electric fields that penetrate deeper into both superficial and subcortical regions, particularly the lateral prefrontal cortex (16).

The repetitive TMS techniques induces antidepressant effects by promoting neuroplasticity in inhibitory neurons (17), yet differences in coil structure and treatment protocol may create variations in the antidepressant effects of these two interventions. According to a meta-analyses, a greater reduction in depressive symptoms has been observed in dTMS with an H1 coil compared to superficial TMS with an F8 coil (18). The underlying physiological mechanisms differentiating these effects, particularly in the context of clinical outcomes, remain underexplored. This gap in psychiatric electrophysiology could be addressed through quantitative electroencephalography (qEEG).

qEEG is widely used to assess TMS-induced effects on brain function, offering insights into real-time brain activity in targeted regions (19, 20) the network-wide impact of TMS (19, 21), and optimization of TMS protocols (19). Moreover, EEG is also used for monitoring the durability of rTMS (22, 23) and dTMS effects over time (24, 25). It allows clinicians to track whether changes in brain activity persist after treatment, providing insight into the long-term efficacy of TMS treatments.

Despite numerous neuroimaging studies investigating the immediate effects of rTMS treatments on depression, there are relatively few qEEG studies examining the long-term effects (≥ 10 session) of rTMS treatments (22, 23) and dTMS (24).

Among these studies, Noda and colleagues explored the impact of 10 session of 20 Hz repetitive TMS on depressive symptoms and cognitive functions in a group of depression patients, applying both clinical scales and resting qEEG before and after the course of rTMS (22) Clinically, they observed significant reductions in Hamilton Depression scores, improved cognitive function measured by the Wisconsin Card Sorting Test after treatment. Electrophysiologically, patients exhibited increased gamma activity, associated with increased GABA-A receptor density and increased in theta-gamma coupling, implicated in memory formation through synaptic plasticity (22). Finally, they found a link between increased gamma activity at left frontal region with the improvement in severity of depression which they interpreted as a candidate biomarkers as to the therapeutic effects of rTMS (22).

Another study investigating resting qEEG after high-frequency TMS treatment for depression at 10 Hz have reported an increase in delta power in the right frontal region with eyes open state, but this was not correlated with clinical improvement (23). On the other hand, another randomized controlled study compared 10-hz rTMS and intermittent theta-burst stimulation (iTBS) showed changes in electrophysiological parameters in stimulation related frequency bands, i.e, alpha band in 10-Hz rTMS and theta band in iTBS, the authors also associated the change in these parameter with clinical improvement for iTBS but not for rTMS (26). However, this two studies which found electrophysiological change without clinical association had been applied 10-hz rTMS on relatively small sample (23), or applied relatively limited rTMS sessions, 10-20 sessions (22, 23).

As for dTMS, a study on 44 patients with MDD investigated qEEG changes after six weeks of high frequency dTMS (24). They used PHQ for measuring clinical symptoms. They found a broad reduction of delta, theta and alpha power in the prefrontal cortex along with decreased power in beta power in the right parietal and occipital lobes. Although they did not find association between the changed prefrontal theta and right frontal alpha activity with clinical improvement, they found the increased delta power in prefrontal regions with treatment response. The authors concluded that TMS may have the potential to improve depressive symptoms by influencing slow-wave brain activity in the prefrontal cortex (24). The authors also conducted another study which integrates both resting state EEG along with Event-Related Potentials, specifically P300 parameters in a group of MDD patients who receive dTMS (25). They found that dTMS responders exhibited reduced delta and beta activity, along with regulated ERP characteristics after treatment (25). Taken together, findings of dTMS studies suggests that dTMS changes neurophysiological states which has a clinical counterpart.

Although there is evidence of a long-term electrophysiological effect of TMS treatments on the brain, the outcomes differ depending on the TMS protocols and coils used. Moreover, not all of the observed electrophysiological changes were found to be associated with the clinical outcome, which highlights the need for further investigation. The objective of this study is to compare the electrophysiological outcomes of rTMS and dTMS on the cortex, and to ascertain whether these outcomes are correlated with clinical improvement. The electrophysiological change was quantified using qEEG, while the improvement in depressive symptoms was assessed using the Hamilton Depression Rating Scale-17 (HDRS-17).

## 2 Method

### 2.1 Study design

This retrospective pre-test post-test study was based on the routinely collected data of the Kemal Arıkan Psychiatry Clinic in Istanbul, Turkey. Each patient had been informed about the procedures and potential side effects of dTMS, and informed consent was obtained. Ethical approval was granted by the local ethics committee (Uskudar University Non-Interventional Ethical Board Decision Number: 61351342/MAY 2022-31). The Population, Exposure, Comparison, Observation (PECO) framework of the study is outlined below:

*Population:* Patients with MDD, aged between 18-60 years, with no response to at least 2 SSRI/SNRI medications.

*Exposure:* 30 sessions of dTMS MDD protocol with H1 coil or 30 sessions of rTMS FDA-approved MDD protocol with F8 coil, applied five days a week for 6 weeks.

*Comparison:* dTMS and rTMS groups were compared with each other (between subject), as well as before and after treatment conditions were compared (within subject).

*Observation:* Resting-state qEEG absolute power, HDRS-17 scores, HARS scores.

Subjects were examined and diagnosed by the same psychiatrist between December 2019 and October 2022. Diagnoses were determined by the DSM-5 (Diagnostic and Statistical Manual of Mental Disorders, Fifth Edition) criteria (27). All the participant data were recruited from the outpatient database. Totally, 60 depression patients aged between 18-60 providing the following criteria were included to the investigations: Non-response to at least 2 SSRI medications, 17-item Hamilton Depression Rating Scale (HDRS) (28) and Hamilton Anxiety Rating Scale (HARS) (29) scores before and after TMS treatments, qEEGs before and after the course of TMS treatments.

**Figure 1:**
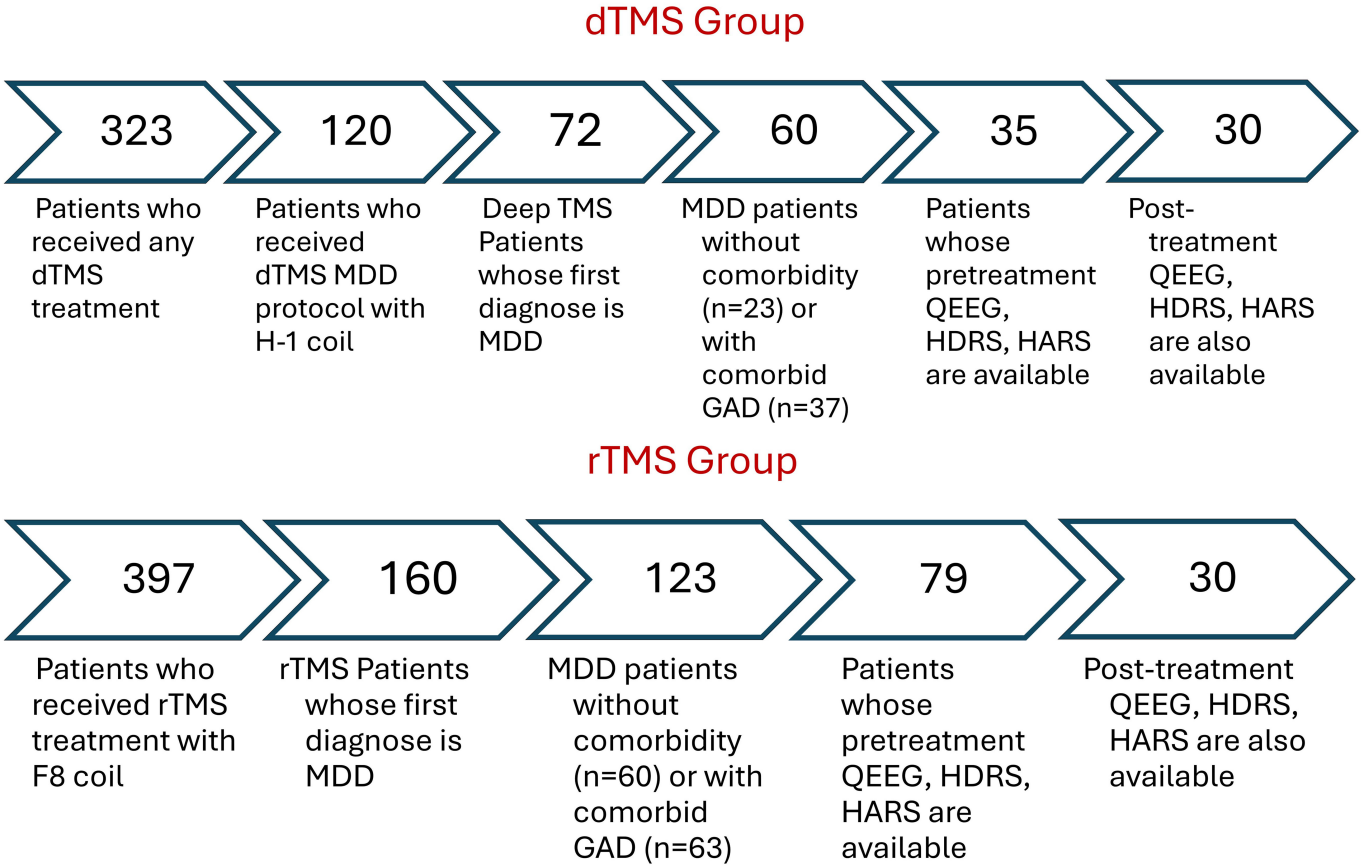
Sample size remained for the analysis after applying inclusion criteria as filters. rTMS, Repetitive Transcranial Magnetic Stimulation with figure-8 coil; TMS, deep Transcranial Magnetic Stimulation with H-1 coil; MDD, Major Depressive Disorder; GAD, Generalized Anxiety Disorder; qEEG, Quantitative Electroencephalography; HDRS, Hamilton Depression Rating Scale; HARS, Hamilton Anxiety Rating Scale.

**Figure 2:**
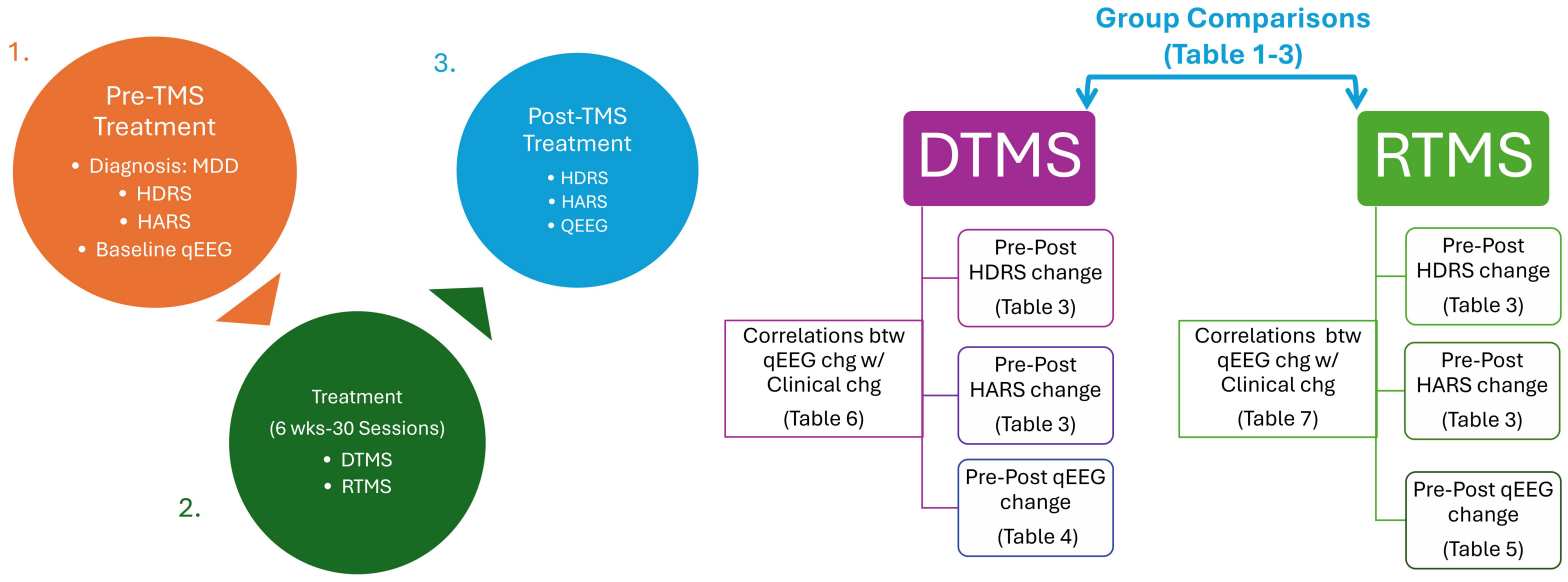
Workflow chart. rTMS, Repetitive Transcranial Magnetic Stimulation with figure-8 coil; TMS, deep Transcranial Magnetic Stimulation with H-1 coil; MDD, Major Depressive Disorder; qEEG, Quantitative Electroencephalography; HDRS, Hamilton Depression Rating Scale; HARS, Hamilton Anxiety Rating Scale.

After applying inclusion criteria, thirty rTMS and 30 dTMS patients were included in the analysis. The steps for reaching that sample size was illustrated in **Figure 1**. Since the patients were clinically symptomatic, they continued their current pharmacotherapy during their TMS treatment. Patients did not take psychotherapy during TMS sessions. None of the participants have acknowledged any neurological and psychiatric comorbidities excluding anxiety. At least a 50% reduction in HDRS after dTMS or rTMS intervention were determined as response criteria. The workflow diagram is depicted in **Figure 2**.

### 2.2 qEEG recording

All subjects underwent qEEG recording before the start of TMS treatment. Resting state qEEG recordings were taped in a silent, dim room with well air-conditioning. A 19-channel (FP1, F7, T3, T5, F3, C3, P3, O1, FZ, CZ, PZ, F4, C4, P4, O2, FP2, F8, T4, and T6) electro-cap was positioned onto the head of the participants in accordance with the 10–20 international system. A transparent electro-gel was injected into the electrodes on the scalp to increase conductivity. The ground electrode was placed in the FPz position. Mastoid electrodes were positioned to both earlobes as reference electrodes. The impedance of electrodes was controlled whether they were <5000 Ω for each electrode. A Neuron-Spectrum-4/P device and programme (30) was utilized to record resting state qEEG activity while patients were in a comfortable sitting-positioned, closed-eye state. The total duration of records was approximately 7 minutes consisted of 3- minute closed-eyed condition, 30-sec open eyes condition, and 3.30-minute closed-eyed condition again. Data were sampled at 500 Hz rate; signals were bandpass filtered at 0.15-70 Hz and notch filtered at 50 Hz.

### 2.3 qEEG analysis

Raw qEEG recordings were stored in European Data Format (EDF). Offline muscle and ocular artifacts were manually removed by an experienced qEEG reader using Neuroguide software (31). Samples with artifacts were deleted and a minimum of 3-minute edited data in closed-eyed condition was obtained. Each patients’ data were averaged across artifact-free 2-sec epochs for each electrode, and the absolute power was computed for the following bands: delta (1-4 Hz), theta (4-7 Hz), alpha (8-12 Hz), alpha1 (8-10 Hz), alpha2 (10-12 Hz), beta (12-25 Hz), beta1 (12-15 Hz), beta2 (15-18 Hz), beta3 (18-25 Hz), high beta (25-30 Hz), gamma (30-50 Hz), gamma1 (30-35 Hz), gamma2 (35-40 Hz), high gamma (40-50 Hz). The calculated data were transferred to SPSS.

### 2.4 Creation of group topographic mapping

The files organized in Neuroguide software were divided into four groups: rTMS pre-treatment, rTMS post-treatment, dTMS pre-treatment and dTMS post-treatment. The edited qEEG files were converted to group qEEG files with the NeuroBatch plugin of the application. Topographic maps including group averages were extracted for each group with the statistical analysis plugin of the application (32).

### 2.5 dTMS intervention

For the dTMS sessions, Brainsway’s H1 coil dTMS System (Brainsway, Har Hotzvim, Jerusalem, Israel) was used. Treatment protocols were applied according to the guidelines of the manufacturing companies.

The measurement of the motor threshold and the intervention were conducted by two certified clinical practitioners in approximately 30-minute sessions. The motor threshold (MT) for depression protocol was determined with the H1 coil positioned on the hand area of the motor strip so that motor activation is induced in the muscles of the right hand. The minimal threshold was defined as the lowest stimulus intensity capable of inducing an observable twitch in any of the fingers. Treatment positions of the coils were located 6 cm anterior to the point where maximum stimulation is observed in the motor threshold measurements.

The duration of dTMS sessions was approximately 25-minutes. In a session, targeted brain areas, left dorsolateral prefrontal cortex of the patients were stimulated by 55 18-Hz train series, each consisted of 36 pulses with a power of 120% of the motor threshold, with 2-sec duration and 20-sec waiting intervals, reaching 1,980 pulses in total. The dTMS treatment protocol for depression was applied according to the study the manufacturer published (33).

### 2.6 Repetitive TMS intervention

For standard TMS sessions, a system titled Figure 8 coil (F-8) from Nuerosoft (Neuro MS/D Therapeutic System, TeleEMG, LLC, Los Angeles, California; 510k no: K160309) is used. Each patient received the FDA-approved TMS protocol for depression. In a session, targeted brain areas, lef dorsolateral prefrontal cortex of the patients were stimulated by 75 10-Hz train series, each consisted of 40 pulses with a power of 120% of the motor threshold, with 2-sec duration and 20-sec waiting intervals, reaching 3000 pulses in total.

The patient lies on the TMS chair. The Figure 8 coil is placed in the hand region of the left motor cortex. The most effective and lowest intensity of stimulation (stimulation threshold) is determined with single shot trials. The head is brought to the left DLPFC region. Stimulation intensity, number of shots, pulse duration, frequency and stimulation threshold parameters are entered to the interface module of the device (34).

### 2.7 Statistical analysis

All the statistical analyses, except qEEG group topographic brain mapping, were computed in SPSS version 24 (35). The significance level was decided to set at p <.05 level for the following analyses.

#### 2.7.1 Normality test

Due to the low sample size, the normality distribution of continuous variables, i.e., scores obtained from clinical scales before and after treatment, percentage reduction in symptom severity, spectral power obtained from qEEG data, difference in qEEG spectral power before and after treatment was assessed using the Shapiro-Wilk test. Normal distribution was achieved in data other than qEEG spectral power. To conform the qEEG data to normal distribution, a logarithmic transformation was applied. As this transformation might result in some data taking negative values and causing issues in calculations, a constant value of 1 was added to the transformed data. However, the absolute power of qEEG in certain electrode-frequency band pairs did not follow a normal distribution in the study sample. Therefore, non-parametric tests were preferred to analyze within-group and between-group differences.

#### 2.7.2 Within and between subject effects

The Wilcoxon signed-rank test was employed to assess changes in absolute power in qEEG bands before and after TMS treatments. The independent samples Mann-Whitney U test was preferred to investigate differences in qEEG absolute powers before dTMS and rTMS treatments for MDD patients. To examine the time-dependent effect of two different types of TMS on qEEG bands, repeated measures analysis of variance (ANOVA) was conducted. In this analysis, measurements before and after TMS treatments were taken as the within-group factor (procedural factor). TMS treatment types (rTMS and dTMS) were selected as the between-group factor.

#### 2.7.3 Clinical variables and correlation with qEEG power change

As the changes in clinical test scores (HDRS and HARS) followed a normal distribution, parametric tests were preferred. Absolute and percentage differences in clinical test scores between TMS treatment groups were measured using independent samples t-tests. The correlation between changes in clinical test scores and qEEG activity was assessed using Pearson correlation test.

## 3 Results

### 3.1 Comparison of demographic and clinical characteristics of the groups

The demographic and clinical data of the participants who were diagnosed with MDD and received either dTMS or rTMS treatment are given in **Tables 1**–**3**. As seen in the table, rTMS and dTMS treatment groups were homogenous in terms of age, gender distribution (**Table 1**), the use of medication, dosage, and the presence of comorbid GAD (**Table 2**), pre-treatment and post-treatment HDRS scores (**Table 3**).

**Table 1:** Comparison of demographic characteristics of the groups.

		Groups		χ^2^	t	p
		rTMS (n=30)	dTMS (n=30)			
**Age(M ± SD)**		35.57 ± 11.90	38.13 ± 13.15		-0.793	0.431
**Gender (n/%)**	**Female**	18/60.0	18/60.0	0.000		1.000
	**Male**	12/40.0	12/40.0			

rTMS, Repetitive Transcranial Magnetic Stimulation with figure-8 coil; dTMS, deep Transcranial Magnetic Stimulation with H-1 coil.

**Table 2:** Comparison of groups based on comorbid generalized anxiety disorder and use of medication.

Variables	rTMS (n/%)	dTMS (n/%)	χ^2^	*p*		
Comorbid GAD	14/46.7	15/50	0.067	0.796		
Drug-free	7/22.6	5/16.7	0.337	0.561		
SSRI	16/53.3	18/60.0	0.435	0.510		
SNRI	6/20	3/10	1.761	0.185		
Antipsychotic	5/16.7	6/20	0.111	0.739		
Anticonvulsant (Lamotrigine)	4/13.3	6/20	0.480	0.488		
Medication doses	Groups	N	M	SD	Means diff.	p
Antidepressants-Fluoxetine dose	rTMS	20	39.4	14.53	3.86	.402
	dTMS	21	35.53	14.98		
Antipsychotics-Chlorpromazine dose	rTMS	5	95	44.72	30	.485
	dTMS	6	125	82.15		
Anticonvulsant-Lamotrigine	rTMS	4	200	0	25	.275
	dTMS	6	175	41.83		

GAD, Generalized Anxiety Disorder; SSRI, Selective Serotonin Reuptake Inhibitors; SNRI, Selective Serotonin Norepinephrine Reuptake Inhibitors; rTMS, Repetitive Transcranial Magnetic Stimulation with figure-8 coil; dTMS, deep Transcranial Magnetic Stimulation with H-1 coil.

**Table 3:** Severity of depression and anxiety of rTMS and dTMS groups before and after treatment.

Variables	Groups	M	SD	Means diff.	p
**Pre-treatment HDRS**	rTMS	23.57	9.07	-0.66	0.774
	dTMS	24.37	8.9		
**Post-treatment HDRS**	rTMS	5.4	6.91	-0.4	0.838
	dTMS	5.87	8.13		
**Pre-post Change HDRS**	rTMS	18.17	10.11	n/A	**< 0.001**
	dTMS	18.5	10.38		**< 0.001**
**Pre-treatment HARS**	rTMS	24.93	12.2	-5.03	0.1
	dTMS	29.27	11.13		
**Post-treatment HARS**	rTMS	4.97	5.01	-1.4	0.389
	dTMS	6.4	1.32		
**Pre-post Change HARS**	rTMS	19.96	11.75	n/A	**< 0.001**
	dTMS	22.867	13.65		**< 0.001**

HDRS, Hamilton Depression Rating Scale; HARS, Hamilton Anxiety Rating Scale; rTMS, Repetitive Transcranial Magnetic Stimulation with figure-8 coil; dTMS, deep Transcranial Magnetic Stimulation with H-1 coil. Bold p values indicate statistically significant results at 0.05 level.

### 3.2 Comparison of pretreatment qEEGs of ındividuals with MDD receiving rTMS and dTMS

The spectral power of pre-treatment qEEGs of individuals with MDD was compared with the non-parametric Mann-Whitney U test. As a result, no statistically significant difference was found in the qEEGs of individuals with MDD receiving rTMS and dTMS before treatment (p > 0.05) (**Supplementary Tables S1–S7**).

### 3.3 Comparison of qEEG absolute powers change within dTMS treatment group

**Figure 3:**
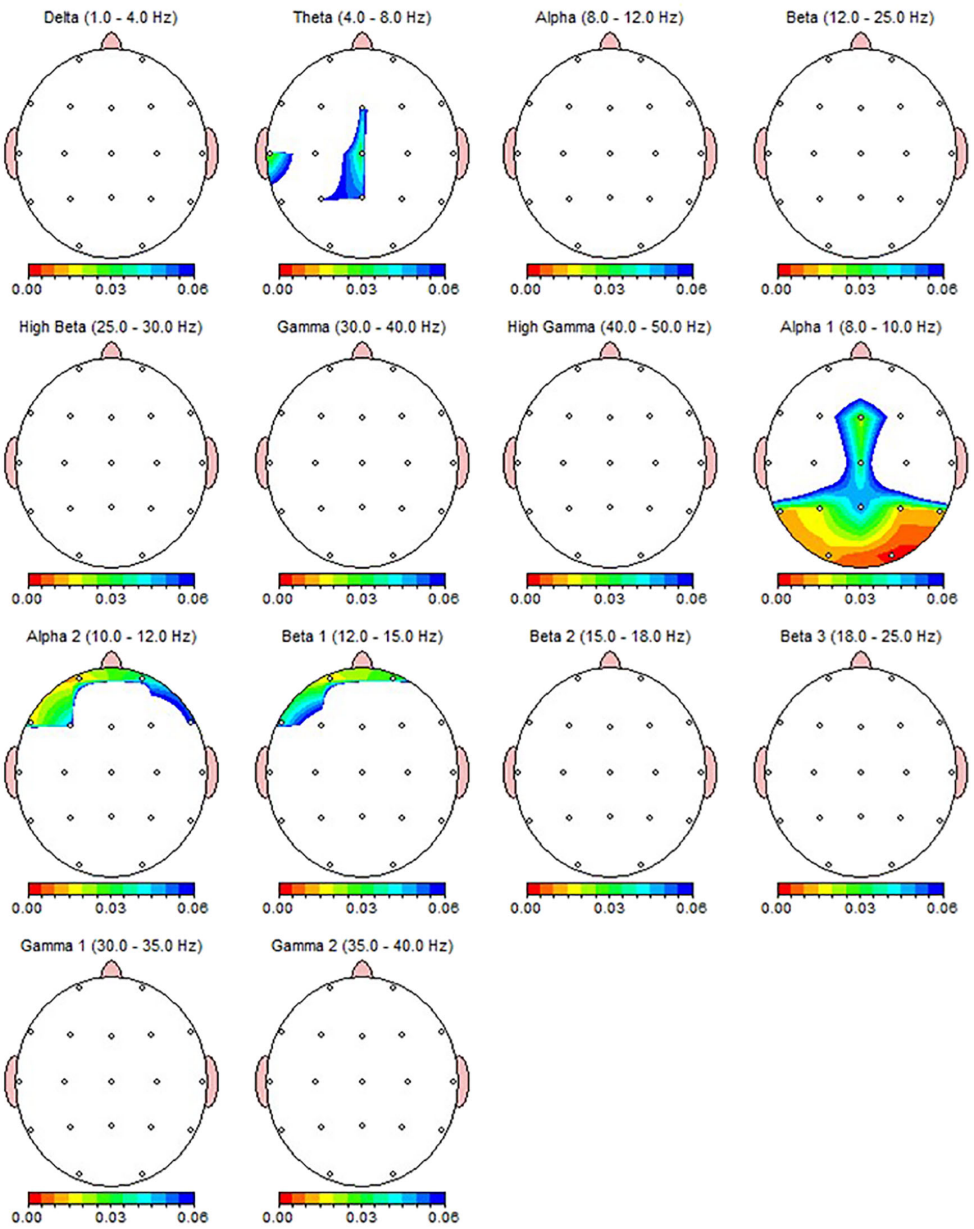
qEEG topographic map of individuals diagnosed with MDD (n = 30) highlighting statistically significant changes from qEEG data before and after 30 sessions of dTMS treatment. On the topographic map, the red regions indicate that the p value obtained in the paired groups t-test is very low and has high statistical significance. Conversely, the blue areas indicate that the p value is borderline (0.05), and the statistical significance is low. The other colors indicate moderate p-value and statistical significance. Although the most significant qEEG changes depicted here were overlapped with the results from non-parametric test (**Table 4**), central theta and prefrontal beta which shown as significant here were not found to be significant in non-parametric results.

Wilcoxon Signed Rank test revealed that patients with MDD exhibited increased Theta power in T3, T5 and alpha-1 power in T5, Fz, P3, P4, O1, O2 region, and decreased alpha-2 power in Fp1 and F7 regions (**Table 4**). Nearly all these results, except T5 theta, were also depicted as significant in qEEG group topographic map comparison which used pair sample t- test (**Figure 3**). On the other hand, the central theta and prefrontal Beta-1 power change which were depicted as significant in topographic map comparison were not found significant in non-parametric test p>.05.

**Table 4:** Statistically significant intra-group differences in relevant qEEG electrode band pairs absolute power before and after dTMS treatment.

Absolute Power	Measurements	Median	IQR-25	IQR-75	p value
**Fz Alpha-1**	Before dTMS	2,335	1,831	2,799	**0.017**
	After dTMS	2,491	1,975	3,061	
**O1 Alpha-1**	Before dTMS	2,528	2,059	3,336	**0.036**
	After dTMS	3,057	2,280	3,944	
**O2 Alpha-1**	Before dTMS	2,659	2,107	3,410	**0.008**
	After dTMS	3,354	2,296	3,913	
**P3 Alpha-1**	Before dTMS	2,431	1,797	2,928	**0.029**
	After dTMS	2,671	2,005	3,529	
**P4 Alpha-1**	Before dTMS	2,615	2,009	3,212	**0.029**
	After dTMS	2,882	2,000	3,483	
**T5 Alpha-1**	Before dTMS	2,134	1,460	2,783	**0.007**
	After dTMS	2,607	1,703	3,186	
**FP1 Alpha-2**	Before dTMS	2,110	1,524	2,753	**0.018**
	After dTMS	1,916	1,637	2,582	
**F7 Alpha-2**	Before dTMS	1,749	1,195	2,245	**0.048**
	After dTMS	1,665	1,313	2,106	
**T3 Theta**	Before dTMS	1,764	1,391	1,994	**0.023**
	After dTMS	2,050	1,553	2,434	
**T5 Theta**	Before dTMS	1,789	1,567	2,452	**0.036**
	After dTMS	2,206	1,596	2,749	

dTMS, deep Transcranial Magnetic Stimulation with H-1 coil. Bold p values indicate statistically significant results at 0.05 level.

### 3.4 Comparison of qEEG absolute powers change within rTMS treatment group

**Figure 4:**
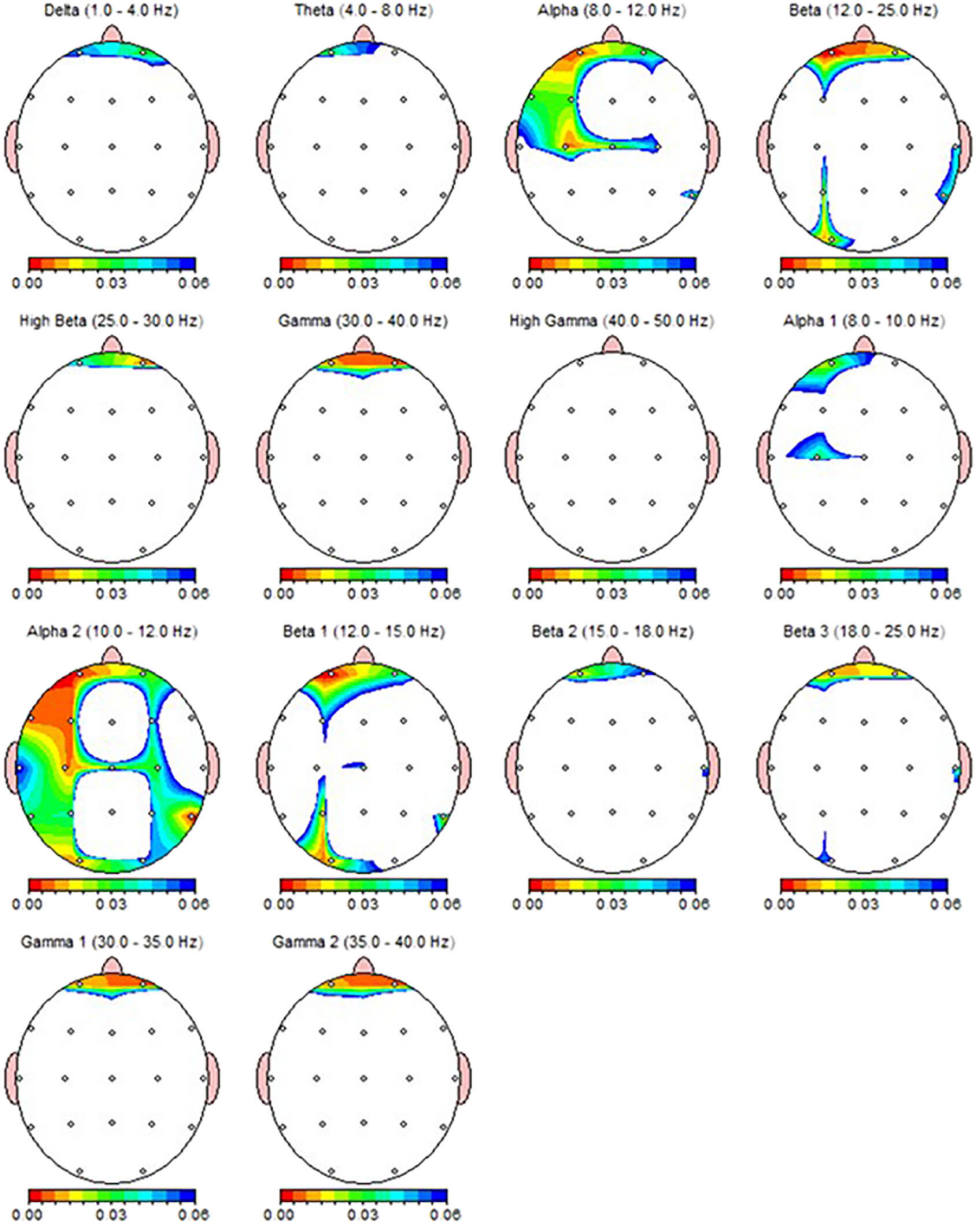
qEEG topographic map of individuals diagnosed with MDD (n = 30) highlighting statistically significant changes from qEEG data before and after 30 sessions of rTMS treatment. On the topographic map, the red regions indicate that the p value obtained as a result of the paired groups t-test is very low and has high statistical significance. Conversely, the blue areas indicate that the p value is borderline (0.05), and the statistical significance is low. The other colors indicate moderate p-value and statistical significance.

According to the Wilcoxon test, a statistically significant decrease was observed in the qEEG spectral power in patients after 30 sessions of rTMS, e.g., prefrontal Alpha, Beta, Gamma, preferably in the left side (**Table 5**). Nearly all the results, except F8 delta and FP2 Beta, were also shown as significant in qEEG group topographic map comparison which used paired sample t-test (**Figure 4**).

**Table 5:** Statistically significant intra-group differences in relevant qEEG electrode band pairs absolute power before and after rTMS treatment.

Absolute Power	Measurements	Median	IQR-25	IQR-75	p value
**F8 Delta**	Before rTMS	3.113	2.787	3.564	**0.015**
	After rTMS	2.996	2.571	3.363	
**C3 Alpha**	Before rTMS	3.064	2.229	3.822	**0.041**
	After rTMS	3.159	2.028	3.598	
**FP1 Alpha**	Before rTMS	2.592	2.062	3.402	**0.018**
	After rTMS	2.699	1.911	3.301	
**C3 Alpha-2**	Before rTMS	2.009	1.541	2.855	**0.028**
	After rTMS	2.054	1.303	2.698	
**C4 Alpha-2**	Before rTMS	2.077	1.425	2.698	**0.033**
	After rTMS	2.043	1.290	2.798	
**F7 Alpha-2**	Before rTMS	1.466	1.175	2.145	**0.018**
	After rTMS	1.465	1.061	1.991	
**FP1 Alpha-2**	Before rTMS	1.739	1.416	2.325	**0.020**
	After rTMS	1.686	1.338	2.261	
**FP2 Alpha-2**	Before rTMS	1.667	1.350	2.357	**0.048**
	After rTMS	1.842	1.197	2.292	
**O1 Alpha-2**	Before rTMS	2.628	1.878	3.628	**0.048**
	After rTMS	2.428	1.899	3.537	
**T5 Alpha-2**	Before rTMS	2.094	1.455	2.990	**0.045**
	After rTMS	2.015	1.140	2.799	
**T6 Alpha-2**	Before rTMS	2.174	1.490	3.303	**0.007**
	After rTMS	1.981	1.216	2.794	
**FP1 Beta**	Before rTMS	2.297	1.907	2.522	**0.003**
	After rTMS	2.208	1.728	2.466	
**FP1 Beta-1**	Before rTMS	1.298	1.121	1.544	**0.003**
	After rTMS	1.234	0.948	1.467	
**FP2 Beta-1**	Before rTMS	1.499	1.162	1.876	**0.041**
	After rTMS	1.450	1.112	1.725	
**FP1 Beta-2**	Before rTMS	1.23	0.88	1.41	**0.031**
	After rTMS	1.06	0.87	1.37	
**FP1 Beta-3**	Before rTMS	1.20	1.01	1.74	**0.039**
	After rTMS	1.22	1.01	1.59	
**FP2 Beta-2**	Before rTMS	1.611	1.141	1.737	**0.013**
	After rTMS	1.421	1.075	1.786	
**FP2 Beta-3**	Before rTMS	1.516	1.323	1.884	**0.028**
	After rTMS	1.583	1.226	1.835	
**FP1 High Beta**	Before rTMS	1.611	1.141	1.737	**0.010**
	After rTMS	1.421	1.075	1.786	
**FP2 High Beta**	Before rTMS	1.516	1.323	1.884	**0.006**
	After rTMS	1.583	1.226	1.835	
**FP1 Gamma**	Before rTMS	0.490	0.279	0.665	**0.013**
	After rTMS	0.342	0.242	0.592	
**FP2 Gamma**	Before rTMS	0.376	0.268	0.513	**0.021**
	After rTMS	0.344	0.262	0.402	
**FP1 Gamma-1**	Before rTMS	0.412	0.233	0.586	**0.018**
	After rTMS	0.298	0.213	0.514	
**FP2 Gamma-1**	Before rTMS	0.328	0.227	0.446	**0.019**
	After rTMS	0.285	0.217	0.346	
**FP1 Gamma-2**	Before rTMS	0.099	0.053	0.174	**0.015**
	After rTMS	0.066	0.044	0.113	
**FP2 Gamma-2**	Before rTMS	0.073	0.053	0.102	**0.011**
	After rTMS	0.066	0.049	0.079	

rTMS, Repetitive Transcranial Magnetic Stimulation with figure-8 coil. Bold p values indicate statistically significant results at 0.05 level.

### 3.5 The relationship between change in clinical symptoms and qEEG change

The relationship between the percentage decrease in HDRS and HARS scores obtained before and after TMS treatments and the change found significant in relevant electrode-band pairs were analyzed by Pearson correlation test. qEEG bands and regions associated with the change in clinical symptoms differed according to the type of TMS treatment (**Tables 6**, **7**). In dTMS treatment, only T5 theta change was associated with the improvement in HDRS and HARS scores (**Table 6**). In rTMS treatment, left frontocentral alpha and right prefrontal beta were associated with HDRS change, while prefrontal beta power change is associated with both HDRS and HARS change (**Table 7**).

**Table 6:** Correlation between the percentage decrease in HDRS and HARS scores and the change in qEEG bands in individuals with MDD receiving dTMS treatment.

	HDRS % Chg	HARS % Chg
HDRS % Chg	1	.833**
HARS % Chg	.833**	1
FZ ALPHA 1	0.102	-0.083
O1 ALPHA 1	-0.009	-0.188
O2 ALPHA 1	0.055	-0.173
P3 ALPHA 1	0.124	-0.100
P4 ALPHA 1	0.259	0.047
T5 ALPHA 1	-0.113	-0.33
F7 ALPHA 2	-0.037	-0.171
FP1 ALPHA 2	0.03	-0.091
T3 THETA	-0.242	-0.223
T5 THETA	-.400*	-.467*

The values at the table represent r values.

** Correlation is significant at the 0.01 level (2-tailed).

* Correlation is significant at the 0.05 level (2-tailed). Bold values indicate statistically significant results at least at 0.05 level.

**Table 7:** Correlation between the percentage decrease in HDRS and HARS scores and the change in qEEG bands in individuals with MDD receiving rTMS treatment.

	HDRS % Chg	HARS % Chg
HDRS % Chg	1	.833**
HARS % Chg	.833**	1
C3 ALPHA	-.396*	-0.115
FP1 ALPHA	-.397*	-0.128
FP1 BETA	-.542**	-.440*
FP1 BETA 2	-.543**	-.418*
FP1 BETA 3	-.552**	-.479**
FP2 BETA 2	-.572**	-.416*
FP2 BETA 3	-.645**	-.447*
FP2 HIGH BETA	-.497**	-0.195
FP2 GAMMA	-.389*	-0.062
FP2 GAMMA 1	-.395*	-0.06

The values at the table represent r values.

** Correlation is significant at the 0.01 level (2-tailed).

* Correlation is significant at the 0.05 level (2-tailed). Bold values indicate statistically significant results at least at 0.05 level.

## 4 Discussion

The present study aimed to compare the two distinct transcranial magnetic stimulation (TMS) modalities approved for treatment-resistant depression, focusing on the electrophysiological changes over the cortex in addition to clinical symptoms.

Consistent with prior expectations based on the literature, a significant reduction in the severity of depression and anxiety symptoms has been observed after the course of both TMS modalities. a randomized sham-controlled study on medication-free patients demonstrated a 24% response rate for active rTMS and 15% for sham conditions after 30 sessions over six weeks (34). In patients receiving concurrent medication and psychotherapy, higher response rates of up to 37.5% were reported for high frequency rTMS (40). Regarding dTMS, a multicenter randomized sham-controlled trial revealed response rates of 38.4% for dTMS versus 21.4% for sham conditions in medication-free treatment-resistant patients following 20 sessions (33). As an adjunct to standard-of-care treatments, a randomized clinical trial reported that both 20 sessions of rTMS with F8 coil (44%) and dTMS with H1 (72%) coil provided better response rates than control group (19%) only continued with pharmacotherapy (36).

In our study, all patients responded to treatment, precluding a formal evaluation of response rates. The high response rates observed, assessed using HDRS and HARS scores, could be attributed to the greater number of TMS sessions and the continuation of standard pharmacotherapy. Additionally, it is plausible that patients with poor initial responses were less likely to complete the full course of treatment. Due to the study design, we were unable to determine the session at which patients began to show clinical improvement; our analysis thus reflects the cumulative impact of 30 sessions.

One hypothesis of the study was that qEEG activity would change in brain regions implicated in the pathophysiology of depression as MDD is characterized by altered electrophysiological activity in subregions of the prefrontal cortex. These changes include increased delta, theta, and alpha activity (associated with depression) and increased beta activity (associated with anxiety). Functional imaging and brain stimulation studies frequently identify hypoactivity in the left dorsolateral prefrontal cortex (DLPFC), which is involved in executive functions, and hyperactivity in the ventromedial prefrontal cortex (vmPFC), associated with self-awareness and emotional regulation (37). Additionally, activity changes extended to the parietal, temporal, and occipital areas. These findings align with studies suggesting that TMS effects may extend beyond the stimulation site, modulating broader neural circuits associated with depression (38).

While simultaneous effects of rTMS on qEEG are well-documented, evidence on the long-term electrophysiological effects of both rTMS and dTMS remains limited. One of them, recording qEEG after 10 Hz rTMS in 31 depression patients, reported a significant increase in gamma power in the left prefrontal region, correlated with a decrease in depressive symptoms (22). Our findings indicate that patients whose gamma activity did not decrease were more likely to achieve better HDRS scores after rTMS treatment, partially supporting the previous study (22).

Another rTMS study conducted by Valiulis and collegues (39) examining the effects of 10 Hz and 1 Hz rTMS on qEEG in MDD patients revealed that frontal alpha asymmetry altered from left to right with low frequency rTMS while high-frequency (10 Hz) rTMS resulted in an increase in delta power in the left hemisphere, an increase in alpha power in the right hemisphere, and an increase in theta power in the parieto-occipital area (39). Similar to that study (39), we also found an increase in alpha power in right hemisphere, yet also decreased alpha and beta activities in the left prefrontal region.

Regarding dTMS results, our findings partially align with a previous qEEG study (24) showing decreased left prefrontal alpha power. However, in contrast, we observed an increase in theta power in the left temporal area and increased alpha power in the right parietal area. This discrepancy may be due to the use of the H1 coil in our study, which targets the left DLPFC, whereas the previous study also employed the H7 coil, targeting the medial PFC.

### 4.1 Limitations

This study has several limitations. The lack of a sham-control group, due to the device’s absence of a sham function, limits the robustness of causal inferences. Furthermore, patients chose their TMS modality based on personal preferences and financial considerations, introducing potential selection bias. The absence of a healthy control group also precluded comparisons with normative electrophysiological data.

Another limitation is the absence of a medication-only control group. Since participants had treatment-resistant depression, clinical changes may reflect either TMS effects or interactions with ongoing pharmacotherapy. Recent studies support this approach, showing that TMS combined with stable medication regimens achieves better response rates than continuing medication alone (36), or starting on a new medication in treatment-resistant depression (40). Overall, it should be noted that this study was a non-controlled study; therefore, the clinical improvement seen after both TMS interventions should be confirmed in future studies in randomized controlled designs.

While the study has various limitations, the study’s strengths include the homogeneity of groups in terms of demographic, clinical, and electrophysiological baseline data, enabling robust comparisons between TMS modalities. While medication use may confound results, similar regimens and dosages across groups mitigate this concern. Additionally, the inclusion of patients with high baseline anxiety levels, while representative of real-world clinical practice, may limit generalizability.

Lastly, when interpreting the study findings, it is essential to consider whether the changing qEEG parameters reflect the immediate physiological effects of TMS or the antidepressant effects of TMS. Nonetheless, areas where changing qEEG parameters were associated with changes in HDRS and HARS scores may be the areas worth further investigation in future studies.

## 5 Conclusion

Our results indicate that several electrophysiological changes have been observed in both TMS modalities, nevertheless there are less qEEG based markers indicating a clinical correlation with such changes, particularly in the case of dTMS treatment. qEEG is a neuroimaging technique that is effective in measuring changes in cortical activity. In this context, it can be predicted that qEEG may provide more markers for rTMS treatment, which reduces symptoms of depression by targeting more superficial and focused areas, in comparison to dTMS, which shows antidepressant effects by focusing on deeper and wider areas of the brain. To ascertain functional change in deep brain regions, future studies may also benefit from utilizing other neuroimaging techniques, such as fMRI and PET, which can demonstrate deeper regions. However, the findings of our study may indicate that qEEG may be a preferable option for electrophysiological monitoring of both TMS treatments in clinical practice, as it is more affordable and provides effective results.

## Data Availability

The raw data supporting the conclusions of this article will be made available by the authors, without undue reservation.
