# Supplementary material for: The effect of repetitive and Deep Transcranial Magnetic Stimulation on quantitative electroencephalography in major depressive disorder
Source: Front Psychiatry. 2025 Jan 6;15:1473743. doi: 10.3389/fpsyt.2024.1473743 (PMC11743562; doi:10.3389/fpsyt.2024.1473743)
Supplement: Supplementary file 1 [file DataSheet1.pdf]

Supplementary Tables

Table S1: Spectral power difference in QEEG Delta band of individuals with MDD receiving rTMS and dTMS before treatment

	Median		
**qEEG Absolute Power**	**rTMS**	**dTMS**	**p value**
C3 Delta	3.026	2.873	0.514
C4 Delta	3.098	2.912	0.379
Cz Delta	3.222	3.104	0.596
F3 Delta	3.190	3.100	0.597
F4 Delta	3.221	3.203	0.505
F7 Delta	3.287	2.991	0.079
F8 Delta	3.113	2.859	0.988
FP1 Delta	3.606	3.735	0.727
FP2 Delta	3.270	3.144	0.716
Fz Delta	3.686	3.763	0.940
O1 Delta	2.846	2.836	0.797

O2 Delta	2.758	2.792	0.203
P3 Delta	2.794	2.778	0.115
P4 Delta	2.829	2.807	0.785
Pz Delta	2.989	3.098	0.514
T3 Delta	2.688	2.491	0.628
T4 Delta	2.539	2.441	0.363
T5 Delta	2.607	2.571	0.565
T6 Delta	2.577	2.764	0.379

Table S2: Spectral power difference in qEEG Theta band of individuals with MDD receiving rTMS and dTMS before treatment

	Median		
**QEEG bandı**	**rTMS**	**dTMS**	**p value**
C3 Theta	2.501	2.359	0.628
C4 Theta	2.412	2.408	0.797
Cz Theta	2.632	2.680	0.940
F3 Theta	2.601	2.485	0.940
F4 Theta	2.662	2.525	0.625
F7 Theta	2.384	2.189	0.649
F8 Theta	2.304	2.093	0.575
FP1 Theta	2.831	2.562	0.192
FP2 Theta	2.697	2.687	0.693
Fz Theta	2.921	2.652	0.832
O1 Theta	2.192	2.074	0.495
O2 Theta	2.165	2.501	0.915

P3 Theta	2.294	2.225	0.231
P4 Theta	2.275	2.404	0.785
Pz Theta	2.418	2.616	0.514
T3 Theta	1.773	1.764	0.628
T4 Theta	1.813	1.730	0.363
T5 Theta	2.043	1.789	0.820
T6 Theta	1.972	2.085	0.596

Table S3. Spectral power difference in qEEG Alpha band of individuals with MDD receiving rTMS and dTMS before treatment

	Median		
**QEEG Bandı**	**rTMS**	**dTMS**	**p value**
C3 Alpha	3.064	2.979	0.891
C4 Alpha	2.862	3.043	0.524
Cz Alpha	3.173	3.307	0.649
F3 Alpha	2.700	3.067	0.355
F4 Alpha	2.714	3.091	0.295
F7 Alpha	2.255	2.528	0.467
F8 Alpha	2.196	2.500	0.275
FP1 Alpha	2.592	2.911	0.262
FP2 Alpha	2.865	3.098	0.208
Fz Alpha	2.485	2.843	0.534
O1 Alpha	3.544	3.491	0.430
O2 Alpha	3.656	3.622	0.379

P3 Alpha	3.176	3.367	0.808
P4 Alpha	3.383	3.449	0.671
Pz Alpha	3.377	3.576	0.467
T3 Alpha	2.143	2.093	0.739
T4 Alpha	1.946	2.016	0.705
T5 Alpha	2.779	2.979	0.879
T6 Alpha	3.110	3.320	0.439

Table S4. Spectral power difference in qEEG Beta band of individuals with MDD receiving rTMS and dTMS before treatment

	Median		
**qEEG Absolute Power**	**rTMS**	**dTMS**	**p value**
C3 Beta	2.506	2.460	0.882
C4 Beta	2.633	2.459	0.657
Cz Beta	2.471	2.560	0.779
F3 Beta	2.528	2.431	0.988.
F4 Beta	2.385	2.461	0.595
F7 Beta	2.226	2.184	0.668
F8 Beta	2.185	1.998	0.882
FP1 Beta	2.297	2.330	0.460
FP2 Beta	2.378	2.384	0.595
Fz Beta	2.271	2.338	0.976
O1 Beta	2.327	2.810	0.174
O2 Beta	2.493	2.929	0.124

P3 Beta	2.434	2.588	0.745
P4 Beta	2.482	2.603	0.322
Pz Beta	2.506	2.701	0.779
T3 Beta	1.975	1.988	0.848
T4 Beta	1.978	1.959	0.564
T5 Beta	2.140	2.174	0.243
T6 Beta	2.223	2.535	0.342

Table S5: Spectral power difference in qEEG High Beta band of individuals with MDD receiving rTMS and dTMS before treatment

	Median		
**qEEG Absolute Power**	**rTMS**	**dTMS**	**p value**
C3 High Beta	1.654	1.755	0.940
C4 High Beta	1.697	1.638	0.903
Cz High Beta	1.637	1.786	0.585
F3 High Beta	1.502	1.643	0.964
F4 High Beta	1.604	1.737	0.976
F7 High Beta	1.491	1.510	0.785
F8 High Beta	1.315	1.423	0.952
FP1 High Beta	1.611	1.582	0.915
FP2 High Beta	1.516	1.653	0.660
Fz High Beta	1.513	1.592	0.976
O1 High Beta	1.487	1.693	0.252
O2 High Beta	1.670	1.761	0.371

P3 High Beta	1.574	1.633	0.524
P4 High Beta	1.602	1.656	0.964
Pz High Beta	1.611	1.770	0.928
T3 High Beta	1.281	1.228	0.534
T4 High Beta	1.281	1.305	0.324
T5 High Beta	1.387	1.429	0.762
T6 High Beta	1.385	1.564	0.649

Table S6. Spectral power difference in qEEG Gamma band of individuals with MDD receiving rTMS and dTMS before treatment.

	Median		
**qEEG Absolute Power**	**rTMS**	**dTMS**	**p value**
C3 Gamma	0.397	0.366	0.701
C4 Gamma	0.331	0.337	0.871
Cz Gamma	0.408	0.364	0.906
F4 Gamma	0.320	0.355	0.802
F3 Gamma	0.385	0.366	0.848
F7 Gamma	0.409	0.348	0.657
F8 Gamma	0.380	0.391	0.882
FP1 Gamma	0.490	0.431	0.894
FP2 Gamma	0.376	0.361	0.848
Fz Gamma	0.488	0.415	0.779
O1 Gamma	0.366	0.402	0.487
O2 Gamma	0.379	0.376	0.965

P3 Gamma	0.370	0.335	0.802
P4 Gamma	0.327	0.303	0.965
Pz Gamma	0.365	0.336	0.813
T3 Gamma	0.374	0.272	0.179
T4 Gamma	0.359	0.298	0.906
T5 Gamma	0.355	0.339	0.657
T6 Gamma	0.352	0.305	0.871

Table S7. Spectral power difference in qEEG High Gammaa band of individuals with MDD receiving rTMS and dTMS before treatment

QEEG ABSOLUTE POWER	Median<br>rTMS	dTMS	p value
C3 High Gamma	0.018	0.016	0.813
C4 High Gamma	0.025	0.018	0.626
Cz High Gamma	0.021	0.020	0.802
F4 High Gamma	0.018	0.022	0.595
F3 High Gamma	0.019	0.022	0.679
F7 High Gamma	0.025	0.019	0.941
F8 High Gamma	0.022	0.017	0.906
FP1 High Gamma	0.026	0.028	0.988
FP2 High Gamma	0.017	0.019	0.813
Fz High Gamma	0.022	0.033	0.701
O1 High Gamma	0.019	0.022	0.416
O2 High Gamma	0.019	0.021	0.976

P3 High Gamma	0.015	0.016	0.679
P4 High Gamma	0.017	0.018	0.877
Pz High Gamma	0.020	0.018	0.848
T3 High Gamma	0.016	0.014	0.657
T4 High Gamma	0.014	0.010	0.767
T5 High Gamma	0.014	0.015	0.615
T6 High Gamma	0.015	0.019	0.535
